# The “Colors” of Moringa: Biotechnological Approaches

**DOI:** 10.3390/plants14152338

**Published:** 2025-07-29

**Authors:** Edgar Yebran Villegas-Vazquez, Juan Ramón Padilla-Mendoza, Mayra Susana Carrillo-Pérez, Rocío Gómez-Cansino, Liliana Altamirano-Garcia, Rocío Cruz Muñoz, Alvaro Diaz-Badillo, Israel López-Reyes, Laura Itzel Quintas-Granados

**Affiliations:** 1 Laboratorio de Farmacogenética, UMIEZ, Facultad de Estudios Superiores Zaragoza, Universidad Nacional Autónoma de México, Batalla 5 de Mayo S/N, Esquina Fuerte de Loreto, Iztapalapa, Ciudad de México C.P. 09230, Mexico; eyebran.villegas@gmail.com; 2 Laboratorio de Reprogramación Celular, Departamento de Fisiología, Facultad de Medicina, Universidad Nacional Autónoma de México, Av. Universidad No. 3000, Coyoacán, Ciudad de México C.P. 04510, Mexico; ibtramon.padilla@outlook.com; 3 Colegio de Ciencias y Humanidades, Plantel Cuautepec, Universidad Autónoma de la Ciudad de México, Av. La Corona No. 320, Colonia Loma La Palma, Ciudad de México C.P. 07160, Mexico; mayra.carrillo@uacm.edu.mx; 4 Colegio de Ciencias y Humanidades, Plantel Casa Libertad, Universidad Autónoma de la Ciudad de México, Calzada Ermita Iztapalapa No. 4163, Colonia Lomas de Zaragoza, Ciudad de México C.P. 09620, Mexico; rocio.gomez.cansino@uacm.edu.mx; 5 Unidad de Estudios Superiores Tultitlán, Universidad Mexiquense del Bicentenario, Av. Ex Hacienda Los Portales S/N, Colonia Villa Esmeralda, Tultitlán C.P. 54910, Mexico; l.altamirano@umb.mx; 6 Laboratorio de Biotecnología, Universidad Politécnica del Valle de México. Av. Mexiquense S/N, Esquina Av. Universidad Politécnica, Colonia Villa Esmeralda, Tultitlán C.P. 54910, Mexico; rocio.cruz@upvm.edu.mx; 7 Department of Health & Behavioral Sciences, Texas A&M University-San Antonio, One University Way, San Antonio, TX 78224, USA; alvaro.diaz@tamusa.edu

**Keywords:** *Moringa oleifera*, sustainable biotechnology, white biotechnology, nanotechnology, green extraction, bioenergy crops, nutraceutical applications, clinical trials, food fortification, regenerative agriculture

## Abstract

*Moringa oleifera* (MO), a nutritionally and pharmacologically potent species, is emerging as a sustainable candidate for applications across bioenergy, agriculture, textiles, pharmaceuticals, and biomedicine. This review explores recent advances in MO-based biotechnologies, highlighting novel extraction methods, green nanotechnology, and clinical trial findings. Although MO’s resilience offers promise for climate-smart agriculture and public health, challenges remain in standardizing cultivation and verifying therapeutic claims. This work underscores MO’s translational potential and the need for integrative, interdisciplinary research. MO is used in advanced materials, like electrospun fibers and biopolymers, showing filtration, antibacterial, anti-inflammatory, and antioxidant properties—important for the biomedical industry and environmental remediation. In textiles, it serves as an eco-friendly alternative for wastewater treatment and yarn sizing. Biotechnological advancements, such as genome sequencing and in vitro culture, enhance traits and metabolite production. MO supports green biotechnology through sustainable agriculture, nanomaterials, and biocomposites. MO shows potential for disease management, immune support, metabolic health, and dental care, but requires further clinical trials for validation. Its resilience is suitable for land restoration and food security in arid areas. AI and deep learning enhance Moringa breeding, allowing for faster, cost-effective development of improved varieties. MO’s diverse applications establish it as a key element for sustainable development in arid regions.

## 1. Introduction

Biotechnology is a broad and rapidly evolving field, often illustrated by a spectrum of colors representing its diverse applications. These “biotechnology colors” help to organize and convey the vast array of scientific, industrial, and societal impacts of biotechnology, from agriculture to medicine and beyond [1].

Several crop cultures have been utilized for their biotechnological potential and economic value. However, *Moringa oleifera* (MO) is unique among biotech crop inputs because it enhances plant growth and yield by improving nutrient uptake (especially phosphorus and nitrogen), shaping beneficial soil microbial communities, and increasing plant stress tolerance through metabolic and lipid changes.

While MO is gaining prominence in sub-Saharan Africa, research on it has not kept pace fully. There is a growing academic interest in MO, particularly in biological and environmental sciences, with most research conducted in South Africa, Nigeria, Egypt, and Ghana. *Moringa* holds significant potential to support food security, climate resilience, and livelihoods in Africa [2]. Despite the potential of *M. oleifera* in agriculture and sustainability, the lack of clinical validation and standardized extraction methods limits its widespread adoption. Therefore, this review aims to synthesize the emerging biotechnological applications of *M. oleifera*, identify key knowledge gaps, and propose future research directions.

### Moringa Genus

The Moringa genus is native to India, Sudan, and Ethiopia, comprising a diverse group of plants with significant nutritional, medicinal, and economic importance [3]. While *M. oleifera* is the most studied, cultivated, and widely used species, other varieties also hold great potential for health and industry. *M. oleifera* is a versatile and sustainable crop utilized in various sectors.

The Moringa genus corresponds to a deciduous tree of the eudicotyledoneae class and belongs to the family Moringaceae. It comprises 13 species of trees, including *M. oleifera*, *M. peregrina*, *M. stenopetala*, and *M. concanensis*, among other species found in tropical regions of Africa and Asia. Each species has unique morphological traits and is adapted to specific local environments in Africa, China, Mexico, and the Middle East [4,5,6].

Despite the abundance of flavonoids, glucosinolates, isothiocyanates, alkaloids, and other nitrogenous compounds in the genus that contribute to its health benefits and commercial potential, there is significant genetic and phytochemical diversity among Moringaceae species [7,8]. For instance, *M. peregrina* exhibits 84% genetic similarity independently, while *M. oleifera* and *M. stenopetala* clusters with 95.3% similarity. Additionally, phytochemical differences among the species are presented in Table 1.

Moreover, the seed extract obtained using n-hexane from MO indicates that the main compounds include 2-decenal (E) (39.14%), 2-undecenal (15.51%), nonanal (3.60%), and 2-octenal (E) (2.48%). By contrast, for *M. peregrina*, the seeds contain 2-decenal (Z) (25.42%), 2-docecen-1-al (9.35%), and 13-docosenoic acid, methyl ester (Z) (4.16%). For *M. stenopetala*, the main compounds are 2-decenal (E) (26.67%), 2-undecenal (24.10%), and nonanal (4.40%) [7]. Genetic and chemical diversity underscores the necessity for conservation and additional research on lesser-known species [6].

The *M. oleifera* genome shows a recent burst of gene duplications from plastids to the nucleus, providing valuable genetic resources for future breeding programs and highlighting the potential of plastid DNA to influence nuclear gene and genome structures. Plastid DNA constitutes 4.71% of the *Moringa* genome, the highest reported in plants [11]. The draft genome of *M. oleifera* was sequenced using the Illumina platform, yielding approximately 231 MB of data that covers around 80% of the genome and identifies over 19,000 protein-coding genes [12,13,14]. This milestone supports future biotechnological advancements. Over the past two decades, reference genomes for over 50 tree species [15,16], including MO, have been published, aiding in identifying the genes related to growth, stress response, and ecological adaptation.

Comparative genomic studies have revealed significant synteny across tree species. A phylogenetic analysis grouped the 50 tree species into three clades, showing that MO is closely related to *Hevea brasiliensis*, a plant that produces latex for rubber, and *Olea europaea* (olive tree) that produces olive oil, from the families Euphorbiaceae and Oleaceae, respectively [17]. The MO germplasm is conserved worldwide and studied for genetic diversity using various markers. To support commercial production, efforts focus on breeding high-yielding, locally adapted varieties and utilizing in vitro propagation [17].

## 2. Current Status of *Moringa oleifera* in the Global Literature over Time

We found 2341 results in the search of PubMed using the words “*Moringa oleifera*”. The oldest article published was “Pterygospermin; the antibacterial principle of *Moringa* pterygosperma, Gaertn” (PMID: 20341204) by R. Raghunandana Rao, Mariam George, and K. M. Pandalai in 1946. The year with the most published articles was 2024 with 320 published articles.

After that, we extended our search to Web of Science (WOS) using the same keywords for searching “*Moringa oleifera*”, limiting the searches from 2000 to 2025. Here, we found 6122 results in the WOS core collection. Analyzing the results, we found that most of the documents are “articles” (5419) followed by “Review Articles” and “Meeting Abstracts” (457 and 174 respectively). We included the division by countries and research themes as a critical component of our bibliometric analysis to provide a global perspective on the development and current status of *M. oleifera* research.

By identifying which countries have made the most contributions, so then “India” covers 20.23% of the publications (1238), followed by China (8.53%) and Egypt (8.33%), we can highlight regional strengths, research priorities, and potential collaborations. This geographic lens also allows us to understand the sociopolitical and ecological contexts in which Moringa research is most advanced, which is particularly relevant for its applications in food security, sustainable agriculture, and public health.

English was the most used language for publications, with 97.8% of the total, followed by the Spanish with 1.11%.

In order to obtain an approximation of the state of the art, we made a bibliometric analysis in VOSviewer version 1.6.19.

The division by themes (e.g., medical applications, water treatment, animal feed, agriculture, biofuel production) emerged from a co-occurrence analysis of keywords using VOSviewer. This thematic clustering helped us organize the vast and interdisciplinary literature into coherent categories that reflect current research trends. Each cluster represents a significant application domain, which we discuss to underscore this review.

The main themes are as follows: (1) medical uses, including antioxidant, anti-inflammatory, and anti-cancer effects; (2) water treatment, utilizing *Moringa* as a natural coagulant, and for pollutant removal; (3) animal feed, improving livestock performance; (4) agricultural enhancement, encouraging plant growth, and enhancing soil quality; and (5) industrial applications, such as oil extraction and biodiesel production.

Based on the bibliometric analysis, we identify the key themes, which are presented in Table 2.

**Figure 1:**
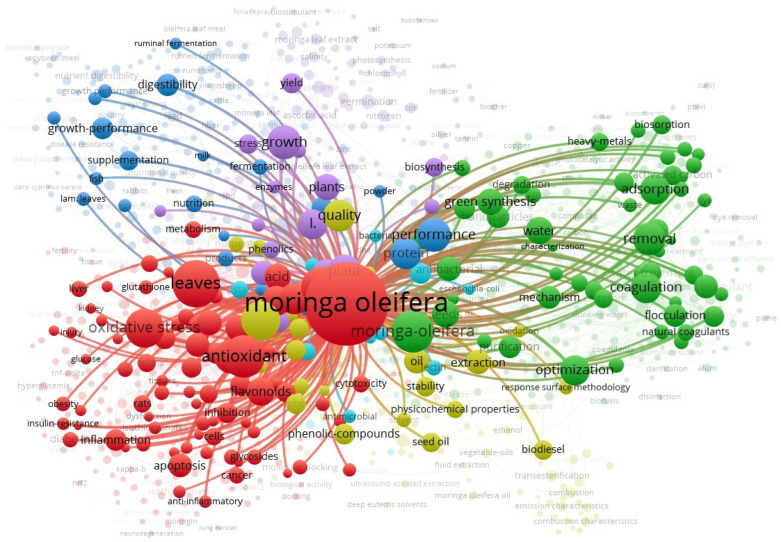
Bibliometric network visualization showing the co-occurrence of keywords related to “*Moringa oleifera*” in the scientific literature. “*Moringa oleifera*” is the central keyword in red, highlighted by its large size and central location. Its prominence and centrality indicate it is the most frequently used term and a hub that connects multiple research themes.

**Figure 2:**
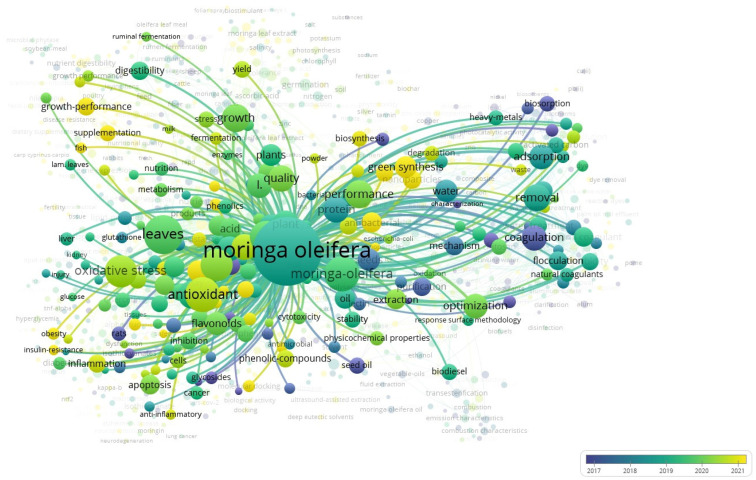
In VOSviewer, the Overlay Visualization expresses additional information by coloring nodes based on a variable or attribute related to the items in the network.

In 2017, *Moringa* research focused primarily on animal applications, with trials conducted on rats. Research was subsequently conducted on the design, optimization, and extraction of various *Moringa* components. In 2021, research began on supplementation, green synthesis, and the biosynthesis of natural compounds. (See Figure 1 and Figure 2).

## 3. Current Application of Moringa in White Biotechnology

White Biotechnology (also called industrial biotechnology) refers to the use of biological systems, like microorganisms and enzymes, to produce chemicals, materials, and energy in an environmentally friendly and sustainable manner. It is primarily applied in industrial processes to create products like biofuels, bioplastics, pharmaceuticals, and food ingredients. The focus is on replacing traditional chemical synthesis with biological methods that are often cleaner, less energy-intensive, and more sustainable [18]. White biotechnology uses biological processes and organisms for industrial applications, emphasizing sustainability and eco-friendliness. *M. oleifera* presents several promising applications in white biotechnology due to its rich bioactive compounds, proteins, polysaccharides, and enzymes [4].

### 3.1. Biofuels and Bioenergy Industries

The depletion of fossil fuels and the urgency to conserve global food resources have accelerated interest in second-generation bioethanol production from non-edible biomass. MO has emerged as a promising biofuel source due to its high seed oil content and lignocellulosic biomass. Techniques such as micro-cutting have been developed to enhance oil yield for biodiesel production, while ongoing research explores MO’s potential for generating biodiesel, biogas, and biohydrogen, along with its socioeconomic and ecological impacts.

MO seeds are rich in bioactive compounds, including hydrolysable carbohydrates (6.5% of seed weight), and neutral sugars, like arabinose and xylose (5%), enhancing their nutritional and industrial value [19]. They contain ~10.13% sugar and ~40% (*w*/*w*) oil, predominantly oleic acid (C18:1) [20,21], making them suitable for biodiesel production. Additionally, the tree’s lignocellulosic biomass can produce second-generation bioethanol, expanding its role in the bioenergy sector [22].

Conventional oil extraction methods include mechanical pressing and Soxhlet extraction, while modern approaches, like aqueous enzymatic, microwave-assisted, ultrasound-assisted, pressurized solvent, and supercritical fluid extraction, preserve phytoconstituents but remain cost-intensive and experimental [23,24,25]. Soxhlet extraction yields vary depending on the solvent: n-hexane (9.3–41.09%) [26,27,28], acetone (25–32.73%) [29,30,31], and chloroform–methanol (41%) [32]. However, Soxhlet extraction is energy-intensive, time-consuming (>7 h), and poses toxicity risks from solvent residues [22,32].

Advances in breeding have produced high-yield MO varieties. The MOMAX3 cultivar introduced by the Advanced Biofuel Center (ABC) yields 2–3 tons of seeds per hectare annually, potentially reaching 8 tons, without genetic modification. Environmental assessments in Australia indicate ~3030 kg of seeds are required for 1000 L of biodiesel, with greenhouse gas emissions higher under irrigated conditions due to increased machinery use. Despite these concerns, MO biodiesel offers significant environmental benefits for mitigating climate change [33]. Sustainability concerns persist regarding land use and food supply competition, though high-yield varieties like MOMAX3 may reduce these risks, albeit with poorly studied ecological impacts.

The 26th UN Climate Change Conference (COP26) in November 2021 reaffirmed the goal of limiting global warming to <1.5 °C, highlighting the need for renewable energy sources. Biomass, including MO, is carbon-neutral, as it absorbs CO_2_ during growth. Pyrolysis of MO seeds—containing 73% carbon, 23% oxygen, and trace minerals like Mg, Al, P, S, K, and Ca—produces fuel and leaf residues usable as fertilizers [33].

Furthermore, MO pod husks, an agricultural byproduct, have been explored for bioethanol production. A novel fungal isolate (*Cladosporium halotolerans* MDP OP903200) demonstrated cellulolytic activity, optimized via response surface methodology. Direct saccharification of *Moringa* pod powder produced 36.06 g/L bioethanol following fermentation with *Saccharomyces cerevisiae*, underscoring the potential of MO residues for sustainable biofuel production [34].

From our point of view, modern extraction methods (microwave-assisted, ultrasound-assisted, supercritical CO_2_) are more efficient than traditional Soxhlet techniques but are still experimental due to high costs and technical issues, like oxidative stability. Our opinion about MO as a second-generation bioethanol source, due to its lignocellulosic biomass, is based on its cultivation in arid regions that support energy production without displacing staple crops. The tree’s carbon-sequestering ability and high seed oil content position it as a climate-smart bioenergy crop. However, environmental trade-offs exist—biodiesel production under irrigation increases greenhouse gas emissions, and the scalability of advanced extraction technologies is currently limited.

### 3.2. Bioprocess Applied to the Extraction of Moringa Phytochemicals

Bioprocessing techniques are increasingly employed to enhance the extraction of valuable phytochemicals from MO, a plant rich in bioactive compounds with applications in food, nutraceutical, pharmaceutical, and cosmetic industries. Submerged fermentation using lactic acid bacteria as a pre-treatment, combined with sonotrode extraction, significantly increases phenolic acids and flavonoids in *Moringa* leaf extracts, improving yields of health-promoting phytochemicals for functional food development [35].

Microwave-assisted extraction (MAE) outperforms traditional methods (e.g., maceration, decoction), especially when optimized for power, temperature, and time, using response surface methodology or the Taguchi method. This approach has achieved yields up to 28.94% and phenolic content of 76.40 mg GAE/g [36,37,38]. Supercritical CO_2_ extraction offers enhanced selectivity and thermal stability for non-polar and low-polar compounds, making it ideal for cosmetic applications and improving bioavailability [39]. Drying methods and solvent polarity also influence phytochemical profiles: air drying preserves more bioactive compounds, while solvent selection determines extraction efficiency [40,41].

**Figure 3:**
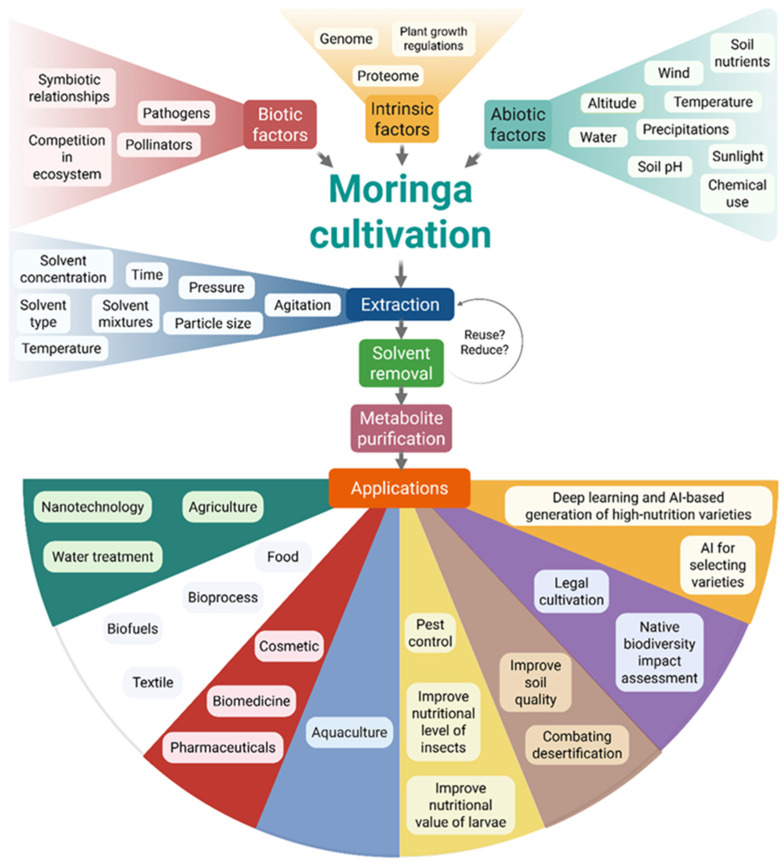
Common bioprocess for extracting phytochemicals from *Moringa*. *Moringa* cultivation thrives when both the intrinsic needs of the plant and external factors (biotic and abiotic) are addressed. Bioprocessing techniques that enable optimal extraction of phytochemical MO are influenced by several factors, including solvent type, time, temperature, and pressure, among others. Solvent removal is a vital step for a sustainable process. The critical step is metabolite purification, which involves several bioprocesses, the parameters of which must be optimized to enhance the yield and suitability of the extracts for various biotechnological applications.

*Moringa* cultivation is shaped by intrinsic, biotic, and abiotic factors (Figure 3). Landrace selection affects stress tolerance (e.g., salinity resilience), while nutrient management (NPK fertilizers, vermicompost), row spacing, and cutting regimes influence biomass and quality [42,43,44,45,46]. Moderate salinity supports growth, and *Moringa*’s adaptability to high temperatures makes it suitable for arid, climate-impacted regions [43,47].

Challenges to cultivation include limited knowledge, poor seedling availability, and pest pressures. Awareness, education, and access to extension services are key to improving adoption and maximizing *Moringa*’s nutritional and economic potential [42,47,48,49].

Advances in fermentation, MAE, and supercritical CO_2_ extraction highlight the importance of optimizing parameters to produce high-value *Moringa*-based products for global food, health, and cosmetic markets.

MO has been incorporated into biopolymer development for biomedical, pharmaceutical, and environmental applications. MO seed powder was integrated into electrospun sodium alginate nanofibers, creating an effective biosorbent for water treatment, and demonstrating significant potential for heavy metal adsorption [50]. Additionally, commercial polyethersulfone membranes modified with MO and graphene oxide (GO), applied via pressurized filtration, showed enhanced selectivity and reduced fouling (<10.55%) during methylene blue dye removal, achieving dye removal efficiencies ranging from 2.85% to 96.73% [51].

MO gum has also been used to produce eco-friendly solid polymer electrolytes, offering improved ionic conductivity, higher amorphousness, and enhanced membrane flexibility due to its low glass transition temperature. These properties confirm MO gum’s viability as a sustainable material for electrolyte applications [52].

From our point of view, the bioprocesses used for MO phytochemical extraction focus on process optimization (solvent type, temperature, pH), but they downplay the importance of green extraction methods that avoid toxic solvents and reduce environmental impact.

### 3.3. Food Industry

MO is increasingly valued in the food industry for its rich nutritional profile and bioactive compounds. Various parts of the plant (leaves, seeds, flowers, pods) are incorporated into functional foods to improve nutritional value, health benefits, and shelf life [53]. MO is rich in proteins, essential amino acids, vitamins (A, B, C), minerals (Ca, Fe, K, Na, P), healthy fatty acids, phenolics, flavonoids, carotenoids, glucosinolates, and functional peptides, conferring antioxidant, anti-inflammatory, antihyperglycemic, antimicrobial, and ACE-inhibitory activities [21,47,54,55,56,57,58,59,60]. MO seed proteins and polysaccharides show antimicrobial, antioxidant, antidiabetic, and antihypertensive effects, making them suitable for food fortification, milk coagulation, thickening agents, and biomedical uses [56,61].

Applications include fortifying bread, yogurt, cheese, soups, beverages, and baked goods, enhancing nutritional quality and phytochemical content often without significantly altering sensory properties [21,54,58,62]. Techniques like ionic gelation encapsulate MO leaf extract in chitosan-coated alginate microbeads to preserve bioactivity as natural antioxidants [63]. MO also supports gut microbiome health, and may help combat malnutrition [21,57,60,62,64]. However, flavor masking agents may be needed to offset sensory changes [65].

Enzymes from MO, such as peroxidase and polyphenol oxidase, exhibit desirable kinetic and physicochemical properties for biotechnological uses [66,67]. MO extracts are applied in active food packaging to extend shelf life and to prevent lipid peroxidation [68]. Additionally, MO leaf extracts effectively inhibit pathogens, like *Escherichia coli*, *Listeria monocytogenes*, and *Salmonella*, in chicken meat, underscoring their potential as natural preservatives [69]. While MO-fortified foods appear safe, more studies are needed to assess long-term consumption effects and impacts on vulnerable populations [62].

From our point of view, MO’s nutrient-rich profile—full of proteins, essential amino acids, vitamins, minerals, phenolics, flavonoids, and glucosinolates—supports its use in functional foods and food fortification. Its antimicrobial properties allow it to serve as a natural preservative, extending the shelf life of products like chicken meat and ground beef. Benefits include improving food security and helping address malnutrition, especially in developing countries. However, challenges, such as its addition to foods, can change sensory properties, like taste and color, requiring extra processing to make it more acceptable to consumers. Long-term safety studies are also missing.

We performed a comparative SWOT table (Strengths, Weaknesses, Opportunities, Threats) for MO in white biotechnology (Table 3).

## 4. Current Application of Moringa in Green Biotechnology

The chemical composition of MO creates synergy in all biotech colors. In the following paragraphs, we describe the application of MO in green biotechnology, including its use in agriculture, the green synthesis of nanoparticles, biocomposites, and environmental remediation.

### 4.1. Agriculture Industry

MO leaf extracts act as natural biostimulants, promoting seed germination, plant growth, yield, nutrient use efficiency, and abiotic stress tolerance (e.g., drought, salinity, heat), while reducing reliance on synthetic fertilizers and pesticides [70,71]. In sorghum, MO leaf extract (MOLE) priming improved germination under salt stress by enhancing antioxidant enzyme activity of superoxide dismutase (SOD), ascorbate peroxidase (APX), polyphenol oxidase (PPO), and catalase (CAT), nitrogen metabolism, and stress-related gene expression, such as HKT-6 (probable cation transporter) and HAK (High-Affinity K+ transporter) genes [72]. Similarly, the foliar application of MO dried leaf extract (MDLE) at 1.5% significantly boosted soybean growth, yield, and seed quality during the reproductive stage [73].

MO extracts exhibit antifungal and antibacterial properties, effectively inhibiting pathogens like *Fusarium*, *Alternaria*, *Rhizoctonia*, and *Botrytis cinerea* [65]. Combined with Fe_3_O_4_ nanoparticles, MO seed extracts demonstrated antibacterial activity in wastewater treatment against *Staphylococcus aureus* [65]. Selenium nanoparticles (SeNPs) synthesized using MOLE showed antibacterial, antioxidant, and plant growth-promoting effects in *Phaseolus vulgaris*, and were effective in dye degradation [74]. Similarly, MOLE and ZnO nanoparticles (MOLE@ZnONPs) mitigated salt stress in *Vicia faba*, enhancing antioxidant defenses and upregulating stress-response genes [75].

Foliar applications of different MOLEs (aqueous, ethanolic, methanolic) improved the growth, yield, and nutritional quality of tomatoes and peppers, with hot water extracts showing the most significant effects on carotenoid and vitamin C content [76].

Micropropagation techniques are employed to overcome limitations in conventional MO cultivation, using plant growth regulators, such as 6-benzlaminopurine (BAP), 1-naphthaleneacetic acid (NAA), indoleacetic acid (IAA), and indole butyric acid (IBA), to optimize shoot and root induction [77]. Stress treatments in MO callus cultures (e.g., salicylic acid, NaCl) also enhance antioxidant metabolite production [65]. For *M. concanensis*, in vitro morphogenesis using cotyledonary nodes achieved high shoot and root regeneration, enabling large-scale propagation with a 60% field survival rate [78].

These findings underscore MO’s potential in sustainable agriculture, biopesticides, phytoremediation, nanoparticle synthesis, and large-scale propagation for food security and pharmaceutical applications.

From our perspective on MO in green biotechnology, MOLE functions as a natural biostimulant and biopesticide, enhancing seed germination, plant growth, stress tolerance (e.g., drought and salinity), and crop yields, while reducing dependence on synthetic agrochemicals. Multiple studies have shown MOLE improves enzymatic activities (e.g., SOD, CAT, PPO) and upregulates stress-responsive genes, indicating molecular-level resilience in crops, such as sorghum, soybean, *Vicia faba*, tomato, and pepper. MO-derived products also exhibit antifungal and antibacterial activity, with root and seed extracts achieving high inhibition rates against phytopathogenic fungi and bacteria. The use of nanoparticles (e.g., SeNPs, ZnONPs) synthesized from MO extracts demonstrates potential in promoting plant growth and mitigating abiotic stress, while also offering eco-friendly solutions for dye degradation in water systems.

### 4.2. Green Nano-Industry

Moringa extracts are utilized to synthesize various nanoparticles (e.g., selenium, iron, silver, carbon nanodots) in an eco-friendly manner, avoiding toxic chemicals [79,80]. Green-produced selenium nanoparticles from MO leaf extract demonstrated strong antibacterial action against *Pseudomonas aeruginosa*, notable antioxidant activity, and effectively degraded crystal violet dye while also influencing *P. vulgaris* growth [74]. Iron nanoparticles synthesized using *M. oleifera* extracts effectively removed 85% of nitrate from water and exhibited antibacterial activity (6 mm of inhibition zones) against *E. coli* [81]. Green synthesized silver nanoparticles from MO leaves exhibited potent antimicrobial activity against *Candida glabrata* and *S. aureus*. They also displayed cytotoxic activities against human melanoma A375 cells at 1000 μg/mL, warranting their biomedical use as antimicrobial and cytotoxic agents [82]. The green synthesis of carbon nanodots (averaging 3.49 nm) using flavonoid extracts from MO leaves is an easy, eco-friendly method with potential for novel heavy metal sensing applications [83]. These nanoparticles exhibit strong antimicrobial, antioxidant, and photocatalytic properties, making them valuable for biomedical applications, water purification, dye degradation, and environmental remediation [79,80]. For example, selenium and iron nanoparticles synthesized using Moringa extracts have been utilized to enhance plant growth, to eliminate nitrates from water, and to inhibit harmful microbes [81].

*M. oleifera* bark waste—rich in phytochemicals— was used for the green synthesis of magnesium oxide nanoparticles (MgO NPs) that enhanced hydrolytic enzyme performance in cellulose digestion. These nanoparticles significantly improved thermal stability and catalytic efficiency when applied to raw fungal cellulases. Notable improvements included sustained filter paper activity (FPA) at 60 °C for 6.5 h, β-glucosidase (BGL) activity for 6 h, and endoglucanase (EG) activity at 50 °C for 4 h with 20 mg MgO NPs. These findings highlight the potential of MO-derived MgO NPs to boost enzyme resilience for industrial bioconversion processes [84].

Our view is that the green synthesis of nanoparticles (e.g., selenium, iron, silver, magnesium oxide) using MO extracts provides sustainable alternatives to chemical methods, creating materials with antimicrobial, antioxidant, and photocatalytic properties. These have uses in agriculture, biomedical applications, and environmental cleanup (e.g., nitrate removal, heavy metal sensing, microplastic capture). Notably, MgO NPs made from Moringa waste improve enzymatic stability for cellulose digestion, adding value to industrial bioconversion processes.

### 4.3. Water Treatment Industry

Moringa seeds and extracts are effective in wastewater treatment, serving as natural coagulants to remove contaminants and to lessen reliance on chemical treatments. Moringa seeds present an environmentally friendly and biodegradable alternative to traditional wastewater treatment methods, providing cost savings, minimizing by-products, and enhancing biodegradability [85]. Carbon nanodots derived from Moringa flavonoids are being developed for heavy metal sensing, providing new tools for environmental monitoring [83].

*M. oleifera* is a renewable and efficient resource in fields such as water purification, emphasizing its effectiveness in removing heavy metals from water, resulting in environmental and socioeconomic benefits, particularly for vulnerable communities facing water scarcity and economic hardship [86]. Moringa seeds serve as natural coagulants and antimicrobials, effectively purifying water and providing an eco-friendly alternative to chemical treatments. *M. oleifera* cationic protein (MOCP) was immobilized on a zeolite substrate to enhance water treatment efficiency. Utilizing central composite design (CCD) for optimization, synthetic turbid water samples (33–67 NTU) were treated with the MOCP-zeolite system. The optimal conditions—12.2 g MOCP/50 mL, 18.5 min mixing time, at 41.3 °C—achieved 61.6% MOCP binding to zeolite and up to 97.43% turbidity reduction, demonstrating the system’s effectiveness for sustainable water purification [87].

Additionally, eco-friendly alternatives to synthetic coagulants for removing microplastics (MPs) from water were developed using MOCP and protein-coated sand (f-sand). Compared to conventional alum and polyacrylamide (PAM), MOCP combined with PAM achieved similar MP removal rates (~70%) in distilled and Mississippi River water. F-sand alone removed about 60% of photo-weathered MPs, while the combination of MOCP with f-sand was less effective, likely due to charge interactions. These findings suggest that MOCP and f-sand are promising, sustainable, and low-impact alternatives for microplastic removal, particularly in natural water systems where synthetic chemicals may pose ecological risks [88].

In addition, *M. oleifera* has several applications in the textile industry, particularly in wastewater treatment and yarn processing. Current research highlights its potential as a natural, cost-effective, and sustainable alternative to conventional chemicals used in textile manufacturing and effluent treatment management. MO seed extracts and by-products are effective natural coagulants for treating textile wastewater. They aid in the removal of color, turbidity, chemical oxygen demand (COD), and other contaminants from effluents. Moringa-based coagulants have demonstrated high efficiency in eliminating dyes and pollutants, sometimes surpassing traditional chemical coagulants like FeCl_3_, particularly in water with high salt content and alkaline pH [89,90]. The process is also more cost-effective than commercial color-removing resins [89].

While Moringa seed powder is effective, studies comparing it with cactus pad powder have found that cactus pads may be more efficient in reducing turbidity, total dissolved solids (TDS), and total suspended solids (TSS) in textile wastewater. However, Moringa still offers significant pollutant removal and remains a viable natural option [72]. Starch extracted from MO seed kernels can be used for sizing warp yarns, providing temporary protection during weaving. Moringa starch offers comparable performance to traditional sizing agents, like cassava and potato starch, when blended with maize starch. Benefits include improved yarn strength, high loom efficiency (95.8%), and easy removal after weaving. Cost analysis indicates substantial savings for textile companies using Moringa starch over conventional chemicals [91,92].

The waste left after oil extraction from Moringa seeds containing water-soluble proteins can be repurposed as a coagulant for dye removal in textile effluents. This approach not only addresses waste management but supports water reuse in textile processes, contributing to a circular economy within the industry [90].

From our perspective, MO seed proteins and by-products are effective natural coagulants for wastewater treatment, removing turbidity, heavy metals, microplastics, and dyes. Innovations, such as MOCP-zeolite composites and protein-coated sand (f-sand), provide eco-friendly alternatives to traditional coagulants, like alum or PAM, supporting circular economy goals. In textiles, MO seed starch and oil-extraction waste contribute to warp yarn sizing and effluent treatment, combining performance with cost savings and environmental advantages.

We performed a comparative SWOT table (Strengths, Weaknesses, Opportunities, Threats) for MO in green biotechnology (Table 4).

## 5. Current Application of Moringa in Red Biotechnology

MO is widely recognized for its diverse biomedical applications due to its rich nutritional profile and abundance of bioactive compounds. It is used in traditional and modern medicine for its antioxidant, anti-inflammatory, antimicrobial, anticancer, antidiabetic, and cardioprotective properties, and is increasingly explored in the fields of pharmaceuticals, nutraceuticals, and nanomedicine.

### 5.1. Pharmaceutical Industry

Moringa extracts and compounds are employed to treat and manage various conditions, including diabetes, hypertension, ulcers, liver and heart diseases, cancer, and inflammation. Its bioactive constituents—such as flavonoids, alkaloids, terpenes, and unique isolates—contribute to these effects by providing antioxidant, anti-inflammatory, and anticancer activities [56,93,94,95,96]. Moringa seed proteins and extracts exhibit antimicrobial, antiviral, and immunomodulatory activities, supporting their application in infection control and immune health [56,97]. MO roots inhibited the production of TNF-α and IL-2, while its pods and seeds suppressed the release of β-hexosaminidase, histamine, IL-4, and TNF-α [98]. Additionally, they also reduced both systolic and diastolic blood pressure and modulated the activity and expression of angiotensin-converting enzyme (ACE) [99,100,101,102].

Moreover, MO leaves lowered cholesterol levels and reduced atherosclerotic plaque formation by 50% and 86%, respectively. They also decreased the protein expression of intercellular adhesion molecule 1 (ICAM-1) and CD55 [103], reduced body temperature [104], prevented memory impairment and cognitive errors [105], and mitigated the harmful effects of electromagnetic and gamma radiation [106,107]. MO flowers and leaves improved bowel movements by regulating stool frequency, weight, and water content, and restored the thickness of colonic muscles and mucus layers [108]. Meanwhile, MO seeds promoted diuresis by increasing both urine volume and concentration [109,110].

Additionally, MO seeds, leaves, and roots downregulated TNF-α and interleukin-1β while improving IL-6 levels [104,111,112,113,114] and reducing tumor weight and progression in sarcoma 180-bearing mice [115]. Leaves and pods also decreased the number of micronucleated peripheral reticulocytes [116,117]. MO leaves and roots enhanced inhibitory neurotransmission by releasing γ-aminobutyric acid (GABA). By contrast, leaves and seeds suppressed acetylcholine release and increased gastric juice volume, while elevating PGE2, IL-10, and GSH levels [118,119,120]. They increased plasma protein concentrations, reduced markers of hepatic dysfunction, and promoted hepatic tissue regeneration [121,122,123]. MO’s leaves, seeds, and roots demonstrated antifungal activity against *Trichophyton rubrum*, *T. mentagrophytes*, *Epidermophyton floccosum*, and *Microsporum canis* [124,125]. Furthermore, MO leaves, seeds, and stems lowered plasma levels of LDL, VLDL, and total cholesterol [126,127,128], while seeds, leaves, and flowers enhanced splenocyte proliferation, activated macrophages, increased nitric oxide production, and elevated white blood cell counts and thymus weight [129,130,131]. Additionally, MO leaves, stems, pods, and seeds demonstrated antibacterial effects against *S. aureus*, *Vibrio cholerae*, and *E. coli* [132].

MO has been studied in clinical trials for its potential health benefits, particularly in metabolic health, immune function, hypertension, and dental applications. While pre-clinical research is extensive, human clinical trials remain limited, but are growing in number and scope. Clinical trials and systematic reviews indicate that MO may help improve glucose control, lipid profiles, and blood pressure, supporting its use in managing metabolic syndrome, diabetes, and cardiovascular risk factors. However, most evidence comes from animal studies, with fewer human trials that are often limited in size or design. Reported human benefits include improved postprandial blood glucose, cholesterol levels, and blood pressure, especially in hypertensive individuals. Although the results are sometimes inconsistent, more robust trials are needed [64,133,134,135,136].

According to the information available (http://www.clinicaltrials.gov, accessed on 2 May 2025), thirty clinical trials have been conducted using *M. oleifera* (Table 5). A double-blind, randomized controlled trial involving HIV-positive adults on antiretroviral therapy found that MO leaf powder supplementation significantly increased CD4 cell counts over six months, indicating immune-boosting potential. However, no significant effects were observed on viral load, weight, or BMI [137]. Moreover, a registered double-blind, randomized controlled trial is investigating whether MO capsules can increase breastmilk volume in early postpartum women, aiming to support exclusive breastfeeding. Results are pending, but the study addresses conflicting data from previous research [138]. Additionally, a randomized controlled trial assessed MO as a natural crosslinker to enhance the durability of dentin bonds in dental restorations. The study found that Moringa pretreatment showed promise in improving clinical outcomes, although the differences with standard care were not statistically significant [139]. Clinical studies have indicated that MO is generally well tolerated, with no significant adverse effects. Some minor gastrointestinal symptoms and changes in appetite or sleep have been observed but, overall, safety profiles are favorable in both normotensive and hypertensive patients [136,140].

Despite promising findings, the number of high-quality, large-scale clinical trials remains limited. Many studies are non-randomized or feature small sample sizes, and there is a need for standardized formulations and dosing. Additional research is required to confirm long-term safety and efficacy, particularly for chronic disease management [133,134,135,140,141].

### 5.2. Biomedical Industry

Moringa gum and polysaccharides serve as pharmaceutical excipients, drug delivery agents, and green polymers in biomedical applications, leveraging their biocompatibility and functional properties [61,142]. Moringa gum and extracts have been incorporated into hydrogels and wound dressings using polymers such as gelatin, chitosan, sodium alginate, and polyvinyl alcohol. These materials are designed for drug delivery, wound healing, and tissue regeneration applications. For instance, the gelatin–chitosan–Moringa biopolymer-based wound dressings exhibit high antibacterial capacities against *S. aureus* and can suppress hemolysis, making them a promising biomaterial for wound care and contamination prevention [143]. Moringa gum-derived hydrogels demonstrate promising biomedical properties, including antibacterial effects and blood compatibility, showing potential for drug delivery and wound dressing applications. Hydrogel dressings gradually released tetracycline, absorbed 7 g of fluid, and exhibited 84% antioxidant activity [144].

Furthermore, sodium alginate-polyvinyl alcohol-MO extracts within sodium alginate/poly(vinyl) alcohol scaffolds exhibit high biocompatibility and remarkable wound healing capacity, with the most significant effect recorded with a 2.5% extract content [145]. Alginate-based films incorporating Moringa powder or essential oil can serve as innovative wound dressings due to their high biocompatibility and non-toxic, non-allergenic properties, making them suitable for potential applications in the biomedical and pharmaceutical industries. Dressings containing 30% Moringa essential oil exhibited 4800% swelling, a tensile strength of 0.248 MPa, and 60.78% antioxidant inhibition [146].

Finally, hydrogels and encapsulation systems utilizing Moringa extracts enable the controlled release of drugs and bioactive compounds, showcasing demonstrated compatibility and gradual release profiles. The green-synthesized biopolymer of polymethyl methacrylate grafted with Moringa gum amphiphilic graft copolymer efficiently releases poorly water-soluble drugs, such as simvastatin and metronidazole benzoate, within 60 min, with a safety profile indicating its potential as a biopolymer for pharmaceutical delivery and tissue re-growth [147]. Moringa enhances biopolymer materials’ tensile strength, flexibility, swelling capacity, and antioxidant activity, which is crucial for wound dressings and other biomedical applications.

Moringa seeds are a rich source of proteins with functional properties suitable for the food and biomedical industries. These proteins can be used as milk coagulants, thickening agents, food and feed ingredients, and drug delivery agents. Moringa seed proteins also demonstrate antimicrobial, antioxidant, and other valuable bioactivities for industrial applications [59]. Proteases extracted from Moringa leaves are effective across various pH levels and temperatures, making them suitable for biotechnological and pharmaceutical applications, such as protein processing and therapeutic uses [148].

### 5.3. Cosmetic Industry

Moringa oil is highly valued in the cosmetic industry due to its light texture and quick absorption into the skin, making it ideal for massage and aromatherapy. Moringa oil extracts are increasingly used in commercial cosmetic products for their skin and hair care benefits. These formulations claim to rejuvenate, nourish, and protect the skin while reducing hair loss. Research from 1999 to 2023 has highlighted a growing interest in MO oil extracts as cosmetic ingredients, with benefits for skin and hair care [149]. However, few studies provide strong bioactivity evidence for MO in topical products. MO seed extract enhances skin hydration and reduces erythema due to its α-tocopherol, sterol, and fatty acid content. It also decreases melanin production, lightening the skin. Seed-based scrub creams tested on rats showed no irritation, and emulsions remained stable at pH 6.8–7.3. MO leaf extract is recognized for its antioxidant and anti-aging properties, with flavonoids and phenolic acids (like quercetin, rutin, and kaempferol) providing photoprotective effects. Sunscreens containing 2–4% MO leaf extract block over 50% of Ultraviolet B radiation (UVB). MO extracts also enhance body washes and protect skin keratinocytes from UVB-induced oxidative stress. Topical treatments with 6% MO stem extract reduced oxidative stress, while MO leaf hydroethanolic and methanolic extracts incorporated into emulsions provided a sun protection factor (SPF) of 2 and improved ultraviolet A radiation (UVA) filter stability [39].

MO seed oil, combined with leaf powder and red rice extract, has been studied for its potential to create a nourishing herbal cream with UV protection and skin brightening properties. The cream, containing 36.4% oleic acid and 0.35% linoleic acid, also includes essential amino acids and ZnO for UV protection. Stability tests showed that the cream maintained its consistency, smell, and color for a month at room temperature, remaining unaffected by environmental factors like sunlight. Additionally, MO seed extract demonstrated skin lightening effects, reducing melanin content by 21–27% in a human epidermis model comparable to kojic acid. The leaf extract also inhibited tyrosinase activity, with luteolin identified as the likely active compound. While the findings are promising, further studies are needed to assess these extracts’ long-term stability, safety, and potential side effects in diverse skin types [65].

Anti-aging effects were demonstrated in human trials where skin roughness, wrinkles, and elasticity improved after three months of using MO leaf cream. MO’s anti-inflammatory properties have been studied in atopic dermatitis models, in which extracts reduce keratinocyte-induced inflammation and increase dermal thickness. Active compounds, such as quercetin, kaempferol glycosides, and isothiocyanates, contribute to its anti-inflammatory effects. However, the stability of these bioactives is sensitive to environmental factors (light, heat, oxygen), prompting the use of encapsulation and microemulsion systems to protect and control bioactive release. Patent data from 1999 to 2023 have indicated a surge in MO-related cosmetic patents, with a plateau from 2016 to 2019 and a decline during the COVID-19 pandemic. The data reflects MO’s significant role in modern cosmetics, with applications in skincare, sunscreens, anti-aging, and anti-inflammatory treatments [39].

MO leaves might be a potential natural inhibitor of the overproduction of tyrosinase, which is linked to hyperpigmentation and fruit browning. The hydro-alcoholic extract of MO leaves exhibited the strongest anti-tyrosinase activity, with an IC_50_ of 98.93 µg/mL, in a dose-dependent manner. The tyrosinase–diphenolase kinetic analysis revealed that the MO extract demonstrated competitive inhibition, while other plant extracts, such as *Ocimum basilicum* and *Artemisia annua*, showed mixed inhibition. The results highlight the compound “rutin” as a promising candidate for use in cosmeceuticals, although in vivo studies are needed to assess its safety and efficacy [150].

## 6. Current Application of Moringa in Blue Biotechnology

Blue biotechnology involves the exploration and use of marine organisms for various human applications, particularly in developing new pharmaceuticals and nutritional supplements to enhance human health. In this context, dietary Moringa extracts have shown the ability to boost immune responses, increase disease resistance, and improve growth in aquaculture species such as whiteleg shrimp. These findings suggest Moringa’s potential as a natural immunostimulant in animal husbandry. A previous report specifically evaluated the effects of Moringa water extract on immune function, disease resistance, and growth performance in whiteleg shrimp [151]. In vitro assays demonstrated that 100–250 ppm of the MO water extract enhanced phenoloxidase activity, phagocytosis, and superoxide anion production. In vivo, shrimp diets supplemented with 2.5 g (ME2.5) and 5.0 g (ME5.0) of Moringa extract per kg significantly improved immune parameters and the expression of several immune-related genes. The ME2.5 group also exhibited better growth and higher survival rates following *Vibrio alginolyticus* infection, particularly after 4 to 7 days of feeding. The 2.5 g/kg dosage proved most effective in promoting shrimp health and resistance [151]. Furthermore, Moringa seed extracts help prevent bacterial growth in treated water, further enhancing their suitability for water purification in marine and coastal communities [152,153].

## 7. Current Application of Moringa in Yellow Biotechnology

MO is widely studied for its applications in insect management and insect nutrition. Its extracts, powders, and oils are used both as natural insecticides and as additives to enhance the nutritional value of edible insects. Moringa seed and leaf powders effectively control major storage pests, such as the cowpea weevil (*Callosobruchus maculatus*), maize weevil (*Sitophilus zeamais*), and khapra beetle (*Trogoderma granarium*), among others. These powders lead to high insect mortality, reduce progeny development, and minimize grain damage and weight loss during storage. Seed powders are generally more potent than leaf powders, and their efficacy increases with higher concentrations and longer exposure times [154,155,156,157,158].

Aqueous extracts and seed oils from Moringa also exhibit strong insecticidal activity, leading to rapid mortality and suppressing reproduction in storage pests. Seed oil can achieve 100% mortality within 24 h, and prevent new infestations [158,159,160]. Moringa powders are repellents, reducing insect infestation in stored grains, such as wheat, flour, and black soybeans. The repellent and toxic effects are attributed to bioactive compounds, like alkaloids, phenolics, and terpenoids [154,157]. Moringa leaf extracts can serve as biopesticides to manage insect pests, such as *Podagrica* spp. on okra (*Abelmoschus esculentus*), thereby reducing pest populations and leaf damage while enhancing crop yield [160].

Furthermore, incorporating Moringa leaves into the diet of *Tenebrio molitor* (mealworm) larvae increases their nutritional value without harming their growth or survival. Mealworms fed Moringa leaves exhibited higher protein, vitamin C, and vitamin A content, making them more nutritious for human consumption [161].

## 8. Current Application of Moringa in Brown Biotechnology

*M. oleifera* and *M. peregrina* are drought-tolerant, fast-growing trees with significant potential for applications in desert and arid environments. Their resilience, nutritional value, and diverse uses make them promising crops for combating desertification, improving food security, and supporting sustainable agriculture in desert regions. For instance, Moringa can be cultivated in semi-arid and arid lands, helping reclaim areas at risk of desertification. Its deep roots and drought tolerance allow it to thrive with minimal water, making it suitable for restoring degraded lands and improving soil quality [162,163]. Therefore, Moringa could help improve food security and nutrition in arid regions because of its high nutritional value and adaptability. Its cultivation boosts local economies and can strengthen the resilience of farming systems in desert environments [2,163].

*M. peregrina*, which is native to desert regions, is valuable for breeding programs aimed at developing crops with better yields and resilience to harsh desert climates. Both *M. oleifera* and *M. peregrina* can be utilized for clonal propagation and hybridization to enhance desirable traits [163,164]. In addition, Moringa extracts serve as natural biostimulants that promote plant growth, increase yield, and enhance stress resistance. They enable crops to withstand salinity, drought, and heat by boosting antioxidant activity and improving water use efficiency, making them valuable for sustainable agriculture in desert regions [71,165]. Moringa seeds are rich in oil, particularly monounsaturated fatty acids, and protein, making them valuable for food, nutrition, and industrial purposes. Moringa oil can be produced in arid regions and has potential for biodiesel production, though cost competitiveness relies on enhancing productivity [162,164,166,167].

## 9. Current Application of Moringa in Violet Biotechnology

MO’s legal status and management can vary significantly depending on the country, particularly where it is considered non-native or potentially invasive. Moringa is not recognized as a native or established crop in South Africa. Under the National Environmental Management: Biodiversity Act (NEM: BA), MO is classified as a Species Under Surveillance for Possible Eradication or Containment Targets (SUSPECT). This designation indicates that Moringa is closely monitored due to concerns about its potential to become invasive and to harm local ecosystems. Consequently, its cultivation is legally restricted and may require active management or eradication in certain contexts [168]. The primary reason for Moringa’s listing under the NEM: BA is its non-native status and associated ecological risks, such as uncontrolled spreading and adverse impacts on native biodiversity. The Act aims to prevent the introduction and proliferation of alien and invasive species that could threaten South Africa’s indigenous flora and fauna [168].

## 10. Current Application of Gold Biotechnology in Moringa Research

Deep learning and artificial intelligence (AI) are applied to Moringa research to accelerate the development of new, nutritionally superior varieties. These technologies predict the qualities of new Moringa plants before they are grown, reducing the time and resources needed for traditional breeding experiments [169,170].

Machine learning and deep learning models are trained on data from parent Moringa varieties, including traits such as plant height, protein content, and potassium levels. These models can predict the characteristics of potential offspring, allowing researchers to identify the most promising crosses without the need for time-consuming field trials. Researchers can bypass several months of traditional nursery and field testing by leveraging AI-driven predictions. This significantly accelerates the development of new Moringa varieties while reducing the required resources. AI also enables more accurate selection of crossbred varieties with desirable traits, such as higher nutritional value, prior to planting. This increases the success rate of breeding programs and supports the cultivation of superior Moringa crops. Overall, integrating machine learning and deep learning into Moringa research facilitates more informed, data-driven decisions, enhancing the efficiency and effectiveness of agricultural research and development [169].

## 11. Biotechnological Challenges

Biotechnological approaches to *M. oleifera* offer significant promise for improving nutrition, health, and industrial applications. However, several challenges must be addressed to realize its full potential. Key challenges include limited genetic resources, insufficient mechanistic understanding, and the need for more empirical evidence, while future directions focus on advanced genomics, breeding, and broader research investment.

Moringa has extensive genetic variability, but the lack of comprehensive germplasm banks and collections of wild and cultivated accessions hinders breeding programs and the development of elite varieties adapted to local conditions. Recent biotechnological advances include genome sequencing, identification of key genes and proteins, and in vitro culture techniques to enhance the production of valuable secondary metabolites. These developments support the improvement of commercially viable traits and elucidate the molecular mechanisms underlying Moringa’s diverse properties.

Furthermore, significant environmental effects on key traits complicate the selection and stabilization of desirable characteristics, such as nutrient content and phytochemical profiles. Many pharmacological claims are based on anecdotal evidence, with limited mechanistic understanding of how bioactive compounds exert their effects. More empirical, mechanistic studies are needed. Most research is preclinical, with few studies on humans, making it challenging to recommend Moringa for disease prevention or treatment.

The structure and activity of bioactive compounds, especially polysaccharides, are highly dependent on extraction and purification methods, which are not yet standardized. The versatility of Moringa-based biopolymers suggests the potential for expanded use in environmental, pharmaceutical, and tissue engineering fields. Research continues to optimize the composition and processing of Moringa-biopolymer blends to maximize encapsulation efficiency, mechanical strength, and functional performance for specific applications.

More studies are needed on the bioavailability, clinical efficacy, and long-term safety of Moringa-derived food products. Standardized experimental designs and further consumer studies are also required to fully realize their potential in the food industry.

## 12. Future Directions for Unlocking the MO Potential

The roadmap for advancing MO research and applications highlights key areas in agrigenomics, clinical standardization, green nanotechnology, and sustainability policy. Although each field shows great promise, targeted strategies are needed to address current challenges and to promote inclusive, evidence-based progress.

### 12.1. Agrigenomics and Breeding

Advancements in genomics-driven breeding—supported by the availability of a draft genome and insights into key transcription factors and metabolic pathways—provide new opportunities for improving MO traits, such as drought tolerance, oil yield, and nutrient density. Tools like CRISPR/Cas genome editing and marker-assisted selection are essential to this effort. However, these methods require well-maintained germplasm banks, high-throughput phenotyping platforms, and strong data-sharing systems. Without adequate investment in research infrastructure, especially in the Global South, where Moringa is native and most beneficial, the goal of doubling seed and oil yield within a decade may be overly optimistic. Therefore, we recommend that international efforts focus on building capacity in genomics and developing open-access, community-curated Moringa germplasm repositories.

### 12.2. Standardization for Clinical Use

Despite MO’s well-documented bioactivity, clinical translation remains limited due to a lack of standardized, large-scale human studies. Future clinical trials should focus on target conditions, such as type 2 diabetes, cardiovascular disease, and cancer, while addressing dosage, formulation, and pharmacokinetics. Standardizing extraction methods and compound profiling is essential to ensure reproducibility and regulatory compliance. Therefore, we suggest forming a global consortium of clinical researchers and pharmacologists to develop standardized Moringa-based interventions and to carry out harmonized multi-center trials, under the guidance of WHO and national regulatory authorities frameworks.

### 12.3. Green Nanotechnology

The use of MO-derived compounds in nanotechnology—such as in drug delivery, biosensing, and antimicrobial systems—is a promising, eco-friendly innovation. However, the field is still in its early stages, with limited translational data. Concerns about biosafety, large-scale production, and long-term stability need systematic attention. We suggest that interdisciplinary collaborations focus on toxicological assessment and scaling up processes for Moringa-based nanomaterials, while ensuring regulatory compliance with existing nanotechnology guidelines.

### 12.4. Policy and Sustainability

MO is well positioned to contribute to climate resilience, degraded land restoration, and circular economy models, especially in arid and semi-arid regions. However, achieving this potential will require coherent policies that support its cultivation, processing, and integration into local and global value chains. There is also a risk that the increasing demand for MO-based products—particularly for pharmaceutical and cosmetic purposes—could result in land-use competition and inequities in benefit distribution. We recommend that national and regional policies support smallholder participation in Moringa value chains, promote agroecological practices, and establish traceability systems to prevent biopiracy and to ensure fair benefit sharing.

### 12.5. Integration Across Disciplines

The progress of MO research will benefit from collaborative efforts across fields such as agronomy, nutrition, pharmacology, and data science. Using artificial intelligence and machine learning for predictive modeling can speed up trait optimization and predicting clinical outcomes. We recommend that funding agencies encourage cross-sector projects that connect agricultural genomics with human health results, while also promoting open data access and ethical use of indigenous knowledge and genetic resources.

## 13. Conclusions

MO stands out as a key crop for sustainable bioenergy, nutrition, and biomedicine. Its high oil-yielding seeds support biodiesel production, while its protein-rich by-products enhance food security and therapeutic applications. Moringa’s adaptability to arid environments, rapid growth, and minimal resource requirements make it ideal for land restoration and climate-resilient agriculture. Its bioactive compounds show promise in managing metabolic disorders, inflammation, and malnutrition, although further clinical validation is essential.

While MO is generally considered safe, long-term studies are necessary to confirm efficacy and to identify any risks, particularly in vulnerable populations. Policymakers should prioritize Moringa in climate adaptation strategies, sustainable agriculture programs, and health initiatives. With targeted research, cross-sector collaboration, and supportive policies, *M. oleifera* could revolutionize global agriculture and healthcare, addressing urgent challenges in nutrition, sustainability, and climate change.

## Figures and Tables

**Table 1:** Phytochemical differences among methanolic extracts of leaves from Moringaceae species.

Moringaceae Species	Total of Phenolic Compounds	Total of Flavonoids	Antioxidant Activity	Ref.
*M. peregrina*	200.12 mg of gallic acid equivalents (GAE)/100 g dry weight (DW)	7 mg quercetin equivalents (QE)/100 g DW	1066.39 mg ascorbic acid equivalents (AAE)/100 g DW	[7]
*M. stenopetala*	243.00 mg GAE/100 g DW	3.05 mg QE/100 g DW	1226.75 mg AAE/100 g DW	[7]
*M. oleifera*	241.05 mg GAE/100 g DW	6.06 mg QE/100 g DW	745.64 mg AAE/100 g DW	[7]
*M. concanensis*	152.81 mg GAE/g ± 3.30 (methanolic leaves)	123.22 mg QE/g ± 6.5 (methanolic leaves)	Strong DPPH/ABTS activity via multiple assays; IC_50_ hydroxyl scavenging ≈ 45.3 µg/mL	[4,9]
*M. ovalifolia*	Not provided	Not provided	Contains antioxidants (e.g., quercetin, kaempferol, myricetin) with confirmed DPPH and ferric reducing activity	[4]
*M. drouhardii*	Minor phenolic content reported	Minor flavonoid content reported	Not provided	[4]
*M. hildebrandtii*	Not provided	Not provided	A non-quantitative phytochemical profile has been reported, including alkaloids, tannins, flavonoids, saponins, steroids, and phenolic compounds.	[10]
*M. longituba*	Not provided	Not provided	Not provided	
*M. ruspoliana*	Not provided	Not provided	Not provided	
*M. pygmaea*	Not provided	Not provided	Not provided	
*M. borziana*	Not provided	Not provided	Not provided	
*M. arborea*	Not provided	Not provided	Not provided	
*M. rivae*	Not provided	Not provided	Not provided	

**Table 2:** Main thematic clusters in MO research.

Color Clusters	Description	Keywords	Focus
Red Cluster	Health and Biomedical Applications	antioxidant, oxidative stress, apoptosis, cancer, inflammation, flavonoids, quercetin	Pharmacological and therapeutic effects of *M. oleifera*, particularly in managing oxidative stress, inflammation, and diseases like cancer and Alzheimer’s.
Green Cluster	Environmental and Water Treatment Applications	adsorption, coagulation, removal, biosorption, flocculation, heavy metals, optimization	Use of *M. oleifera* as a natural coagulant or biosorbent for water purification and environmental cleanup.
Blue Cluster	Animal Nutrition and Feed	growth-performance, digestibility, fermentation, supplementation, metabolism, sheep, goats	Application of *M. oleifera* in livestock nutrition, enhancing animal growth, digestion, and health.
Purple Cluster	Agricultural and Plant-Based Research	germination, photosynthesis, biomass, biosynthesis, yield, phytohormones, quality	*M. oleifera* in plant growth, productivity, and sustainable agriculture.
Yellow Cluster	Oil Extraction and Biofuel	seed oil, biodiesel, transesterification, extraction, stability	Industrial and biochemical extraction of oil from *M. oleifera* seeds for use in biodiesel and bio-based industries.

**Table 3:** Strengths, Weaknesses, Opportunities, Threats for MO in white biotechnology.

Strengths (S)	Weaknesses (W)	Opportunities (O)	Threats (T)
Rich in bioactive compounds, such as proteins, flavonoids, phenolics, and enzymes, making it suitable for various industrial applications.	Advanced extraction techniques, like supercritical CO_2_ and MAE, are expensive and currently not suitable for large-scale manufacturing.	Growing demand for eco-friendly and carbon-neutral energy sources like biofuels and bioplastics.	Environmental trade-offs of large-scale monoculture plantations, such as biodiversity loss and soil depletion.
Seeds with high oil content (40% *w*/*w*) are suitable for biodiesel production, and lignocellulosic biomass can be used for bioethanol.	Traditional techniques, such as Soxhlet extraction, require a lot of solvents, take a long time, and pose environmental concerns.	Development of high-yield cultivars like MOMAX3 and bioengineered enzymes to optimize bioprocesses.	High production costs and technological barriers hinder the transition to industrial-scale operations.
Suitable for arid climates and poor soils; ideal for areas affected by climate change.	There is limited quantitative data available for non-oleifera Moringa species, and long-term toxicology studies are insufficient.	Growth in the functional food and nutraceutical sector driven by Moringa’s health benefits.	Regulatory challenges for new food ingredients, particularly in affluent nations.
Applications are diverse, encompassing biofuels, nutraceuticals, biopolymers, and water purification systems.	Variation in yield and quality is caused by environmental and farming factors.	Utilized water purification membranes and biopolymer-based packaging to promote environmental sustainability.	Socioeconomic challenges include a lack of awareness, limited access to planting materials, and the marginalization of smallholders.
Potential use as a natural preservative and food fortifier to fight against malnutrition.	Incorporating sensory issues like taste and color changes into food requires extra processing.	Potential for circular bioeconomy initiatives utilizing agricultural residues, such as pod husks for bioethanol production.	Yield may still be influenced by climate variability, even with drought tolerance.

**Table 4:** Strengths, Weaknesses, Opportunities, Threats for MO in green biotechnology.

Strengths (S)	Weaknesses (W)	Opportunities (O)	Threats (T)
MO’s broad-spectrum bioactivity and compatibility with sustainable practices establish it as a crucial agent for green biotechnology and the bioeconomy. Its multifunctionality—from soil enhancer to nanoparticle precursor—makes it especially relevant for integrated systems (e.g., agriculture and water treatment).	Most applications are still in the experimental stage, especially nanoparticle synthesis and bioprocess scaling. Cost-effectiveness and standardization remain major obstacles. Changes in food sensory properties and environmental trade-offs (such as large-scale monocultures) are not fully addressed.	Advancing bioprocess optimization, integrating MO into circular economy models, and improving knowledge transfer to smallholders could maximize impact.	Regulatory frameworks for nanoparticle use, potential allergenicity or toxicity in food systems, and scalability of in vitro propagation are essential issues requiring further research.

**Table 5:** Clinical trials for *Moringa oleifera*.

Study Title	NCT Number	Locations	Study Status	Sex	Age	Phases	Study Type	Conditions	Summary
Effect of *Moringa oleifera* mouthwash	NCT05191069	Islamabad Capital Territory, Pakistan	Unknown *	All	Child, adult	NA	Interventional	Orthodontic appliance complication	This study evaluates the effectiveness of MO mouthwash in enhancing oral hygiene during orthodontic treatment. It examines its role in preventing gingivitis, periodontitis, plaque formation, enamel demineralization, tooth discoloration, and reducing the bacterial load in plaque.
*Moringa oleifera* on bone density	NCT03026660	Boone, North Carolina, United States	Completed	Female	Adult, older adult	NA	Interventional	Osteoporosis, osteopenia, postmenopausal osteoporosis	This study aims to evaluate the effects of daily 1000 mg MO supplementation over 12 weeks on bone structure and function in postmenopausal women.
*Moringa oleifera*–antiretroviral pharmacokinetic drug interaction	NCT01410058	Harare, Zimbabwe	Completed	All	Adult, older adult	-	Observational	HIV	The use of MO Lam leaf powder at its traditional dosage did not significantly affect the steady-state pharmacokinetics of nevirapine.
Moringa supplementation for improved milk output	NCT05333939	Lexington, Kentucky, United States	Completed	All	Child, adult, older adult	NA	Interventional	Breastfeeding	This study gathers data on whether daily 4 g MO supplementation for four weeks enhances breast milk quantity, quality, and infant health versus placebo. Moringa is expected to boost milk output and the proportion of the infant’s intake from the mother.
Effect of *Moringa oleifera* infusion on health	NCT04314258	Moka, Mauritius	Unknown *	All	Adult, older adult	NA	Interventional	Metabolic syndrome	This study explores the effects of MO leaf tea on health markers in hyperglycemic individuals (fasting blood glucose ≥ 5.5 mmol/L). Objectives include assessing impacts on blood glucose, lipid profiles, and antioxidant levels, comparing healthy and hyperglycemic individuals.
Effect of *Moringa oleifera* leaves on glycemic control of women with type 2 diabetes	NCT06517602	Tindouf, Algeria	Completed	Female	Adult, older adult	NA	Interventional	Type 2 diabetes	This clinical trial assessed if daily MO leaf powder supplementation, alongside oral hypoglycemic therapy, improved glycemic control in Sahrawi women with type 2 diabetes. Researchers measured changes in glycosylated hemoglobin, fasting blood glucose, and clinical, metabolic, and body composition parameters at the study’s start and end.
Remineralization efficacy of *Moringa oleifera* varnish vs MI varnish in initial carious lesions over 6-month follow up: a randomized controlled clinical trial	NCT06905379	Cairo, Egypt	Not yet recruiting	All	Adult	NA	Interventional	White spot lesions [initial caries] on smooth surface of tooth	This clinical trial assessed the remineralization efficacy of MO varnish versus MI Varnish (CPP-ACP) on incipient carious lesions. Participants aged 25–35 with at least one active white spot lesion (WSL) and good oral hygiene provided informed consent
Effects of *Moringa oleifera* on hsCRP and Hgba1c level of patients in Hospital ng Maynila medical center diabetic clinic	NCT02308683	Location not provided	Completed	All	Adult, older adult	Phase 1	Interventional	Diabetes	This cohort study investigates the effects of MO leaf supplementation on inflammation and glycemic control in patients with type 2 diabetes. This study focuses on high-sensitivity C-reactive protein (hsCRP) as a key inflammatory marker, along with HbA1c. clinical outcomes.
Effect of Moringa leaf extract on disease activity in rheumatoid arthritis patients	NCT05665985	Surakarta, Central Java, Indonesia	Completed	Female	Adult	Phase 1, phase 2	Interventional	Rheumatoid arthritis	This study evaluated the effects of MO extract on rheumatoid arthritis activity. Patients received MO in a 30-day treatment regimen to assess changes in disease activity during the intervention.
Effect of aerobic training and *Moringa oleifera* on dyslipidemia and cardiac endurance	NCT04164771	Location not provided	Unknown *	Male	Adult	NA	Interventional	Dyslipidemias	Moringa leaves are highly effective against various diseases, particularly diabetes, blood pressure issues, dyslipidemia, and cancer.
Effect of *Moringa oleifera* on metformin plasma level in type 2 diabetes mellitus patients	NCT03189407	Location not provided	Completed	All	Adult, older adult	NA	Interventional	Type 2 diabetes mellitus	This study evaluated the effects of a seven-day, twice-daily hot water infusion of dried MO leaves on the plasma concentrations of Metformin in type 2 diabetes patients already on Metformin for at least three years months.
*Moringa oleifera* (drumstick leaves) for improving hemoglobin, vitamin A status and underweight among adolescent girls in rural Bangladesh: a quasi-experimental study	NCT04156321	Dhaka, Bangladesh	Unknown *	Female	Child	Phase 3	Interventional	Assess the impact of Moringa leaves on serum hemoglobin and vitamin A level among the adolescent girls	NA
Anticariogenic effect of *Moringa oleifera* mouthwash compared to chlorhexidine mouthwash	NCT04575948	Location not provided	Not yet recruiting	All	Adult	Phase 2, phase 3	Interventional	Plaque, dental, antimicrobial, mouthwash, cytotoxicity	Part I: This in vitro study aims to formulate a non-toxic mouthwash from MO leaf extract, which has antimicrobial activity, for use in Part II. Additionally, the mouthwash’s stability and efficacy will be evaluated. Part II: This randomized controlled trial assesses the antibacterial, antiplaque, and anticariogenic effects of MO mouthwash versus chlorhexidine mouthwash.
Effects of *Moringa oleifera* leaves on glycemia, lipemia, and inflammatory profile in prediabetic patients	NCT04734132	Madrid, Spain	Completed	All	Adult, older adult	NA	Interventional	Prediabetes	This proposal studies the efficacy of MO in controlling glycaemia in prediabetic subjects. A 3-month dietary intervention with MO dry leaf capsules will be compared to a placebo.
Nutritional impact of *Moringa oleifera* leaf supplementation in mothers and children	NCT04587271	Kisumu, Kenya	Completed	All	Child, adult, older adult	NA	Interventional	Malnutrition, wasting, and growth failure	The primary outcomes were infant growth and maternal milk production, while secondary outcomes included maternal and infant vitamin A and iron status and changes in their intestinal health.
Effects of *Allium sativum* and *Moringa oleifera* extract on dental enamel	NCT05744752	Karachi, Sindh, Pakistan	Unknown *	Male	Child	NA	Interventional	Lead poisoning	The objective is to compare the protective effects of *Allium sativum* (AS) and MO on dental enamel defects from lead and to determine their benefits in remineralizing dental enamel.
Effect of *Moringa oleifera* leaf on hemoglobin levels in anemia	NCT05737862	Bandung, West Java, Indonesia	Completed	Female	Child, adult	Phase 3	Interventional	Anemia of pregnancy	This study aimed to compare hemoglobin levels in pregnant women between the treatment group, which received Moringa leaf capsules and iron tablets, and the control group, which received only iron tablets.
Evaluation of *Artemisia annua* and Moringa	NCT03366922	Mbarara, South Western, Uganda	Completed	All	Adult, older adult	NA	Interventional	HIV infections	Determine the effect of *A. annua* L. and MO leaf powder on CD4 cell count and immunological indices in HIV patients receiving highly active antiretroviral therapy.
Anti-plaque and anti-gingivitis effects of Moringa plant extract and fluoride toothpastes	NCT05390099	Giza, Egypt	Unknown *	All	Child	NA	Interventional	Oral disease	This study assesses and compares the anti-plaque and anti-gingivitis effects of Moringa plant extract and fluoride toothpastes in Egyptian children.
Effect of *Moringa oleifera* capsule in increasing breast milk volume in early postpartum patients	NCT04487613	Bangkok, Thailand	Completed	Female	Adult, older adult	Phase 4	Interventional	Postpartum women	This study aims to assess how MO leaf capsules influence breast milk production.
Effects of *Moringa oleifera* leaf powders on hematological profiles in pregnant women with iron deficiency anemia	NCT06875947	Cianjur, West Java, Indonesia	Not yet recruiting	Female	Adult	Phase 4	Interventional	Iron deficiency anemia of pregnancy, pregnancy complications, inflammation, *Moringa oleifera*, cytokines (IL-1, IL-6), hepcidin	This study investigates micronized Moringa leaf powders as a natural supplement to enhance hemoglobin levels in pregnant women with iron deficiency anemia. Participants will undergo regular blood tests to assess hemoglobin levels, iron status markers (hepcidin, TIBC), and inflammatory cytokines (IL-1, IL-6). This study also evaluates the safety of Moringa supplements, focusing on liver and kidney functions.
Impact of dried *Moringa oleifera* leaves in enhancing hemoglobin status	NCT03514472	Location not provided	Completed	Female	Child, adult	NA	Interventional	Anemia, iron deficiency	This research project targets nutritional deficiencies, particularly iron deficiency anemia in reproductive-aged females from underprivileged groups. Anemia can result in stillbirths, preterm deliveries, and low birth weight, potentially leading to cognitive disabilities, emphasizing the need for priority treatment.
Effect of Moringa leaf capsules on glycemic control of type 2 diabetic patients	NCT06125873	Islamabad, Federal, Pakistan	Enrolling by invitation	All	Adult, older adult	Phase 2	Interventional	Diabetes mellitus type 2	A clinical trial will involve 50 patients randomly divided into two groups to compare glycemic control in Type 2 diabetes mellitus using MO capsules.
Evaluation of *Moringa oleifera* leaf extract versus sodium hypochlorite in pulpectomy of nonvital primary molars	NCT06948526	El-Manial, Giza, Egypt	Not yet recruiting	All	Child	NA	Interventional	Nonvital primary molars	This trial compares the success of MO leaf extract and sodium hypochlorite as intracanal irrigants in pulpectomy of nonvital primary molars in children aged 3 to 7. It evaluates clinical parameters (pain, swelling, mobility) and radiographic healing (periapical changes, root resorption) over 12 months.
Antifungal potential of *Moringa oleifera* against otomycosis	NCT04768829	Minya, Egypt	Completed	All	Adult	Early phase 1	Interventional	Otomycosis	One group of patients with otomycosis received Nystatin ear drops, while the other received Moringa ear drops. An otolaryngologist performs an endoscopic examination, and their swabs will be analyzed using ELISA assays.
A study to explore the effect of *Moringa oleifera* (E-HS-01) on flow mediated dilatation and hemodynamics	NCT05002881	Mumbai, Maharashtra, India	Unknown *	Male	Adult	NA	Interventional	Endothelial function	This study evaluates how MO affects vascular endothelial function, investigating its vasodilation potential by analyzing flow-mediated dilation (FMD) and blood flow velocity (BFV) in healthy males.
Effect of *Moringa oleifera* leaf extract on postoperative pain and bacterial reduction in mandibular premolars	NCT05348824	Location not provided	Unknown *	All	Adult	Phase 2, phase 3	Interventional	Necrotic pulp	This study clinically compares post-operative pain intensity and bacterial reduction with MO leaf extract solution versus 2.5% NaOCl in asymptomatic necrotic mandibular premolars treated in a single visit.
The cardiovascular and renal effects of *Moringa oleifera* extracts and *Stevia rebaudiana* Bertoni in patients with type 2 diabetes mellitus	NCT04254029	Yaoundé, Cameroon	Completed	All	Adult, older adult	Phase 4	Interventional	Benefits of capsules of *M. oleifera* and *Stevia rebaudiana* Bertoni in patients with type 2 diabetes mellitus before and after 45 days of add-on therapy	This study aimed to evaluate MO and stevia’s cardiovascular and renal benefits in type 2 diabetes patients over 8 weeks.
Antidiabetic potential of Moringa and Dom extract	NCT05898750	Minya, Egypt	Completed	All	Adult	Early phase 1	Interventional	Diabetes	The antidiabetic properties of *Hyphaene thebaica* fruits and MO leaves will be studied in type 2 diabetic patients consuming tea from both for six weeks. Their fasting blood glucose levels will be monitored daily, alongside other biomarkers, such as insulin concentration, lipid profile, liver enzymes, c-peptide, and glycated hemoglobin.
Moringa; delivering nutrition and economic value to the people of Malawi	NCT04092517	Aberdeen, United Kingdom	Completed	All	Adult, older adult	NA	Interventional	Malnourishment	This study compares Moringa as a substitute in supplementary foods to evaluate nutrient bioavailability, bioactivity, and the plant’s activities. It assesses Moringa’s potential as an economically viable crop to support a resilient food supply chain in Malawi, ensuring access to essential nutrients.

* The study has passed its completion date, and the status has not been verified in more than two years. NA: not available. NCT Number: ClinicalTrials.gov Identifier.

## Data Availability

The datasets generated in this study are available from the corresponding author upon reasonable request.
